# Prevalence and associated factors of developing venous thromboembolism in the perioperative period

**DOI:** 10.1186/2197-425X-3-S1-A231

**Published:** 2015-10-01

**Authors:** K Okada, N Saito, H Matsumoto, C Kim, N Hato

**Affiliations:** Nippon Medical School, Shock and Trauma Center, Inzai, Japan; Department of Anesthesiology, Nippon Medical School, Inzai, Japan; Division of Intensive Care Unit, Nippon Medical School, Inzai, Japan

## Intr

Previous studies suggested that the prevalence of venous thromboembolism (VTE) is considerably lower in Asian populations compared with Western populations. It is therefore indicated that the incidence and risk of developing VTE among Asian countries should be evaluated separately.

## Objectives

This study aimed to clarify the epidemiology and associated factors of developing VTE during the perioperative period in a tertiary care hospital in Japan.

## Methods

We retrospectively reviewed patients who underwent operation under general anaesthesia between August 2011 and December 2013. VTE in the perioperative period was identified through contrast enhanced CT which was performed from 30 days before operations until 90 days after operations. Multivariate logistic regression was conducted to assess the associated factors of developing VTE.

## Results

8287 patients were included in this analysis. The mean age of subjects was 58.7 ± 16.5 years and there were 3973 (47.9%) female patients. 102 (1.23%) cases showed the evidence of VTE during the period and 64 were diagnosed postoperatively. Of these, 34 (53%) patients received pharmacological prophylaxis after operation. In-hospital mortality of patients with VTE was 3.9%. Multivariate analysis revealed that associated factors of developing VTE were female patients (odds ratio [OR] 3.24, 95% confidence interval [CI] 1.84 to 5.70, P < 0.001), receiving open reduction internal fixation (ORIF) (OR 2.96, 95%CI 1.49 to 5.88, P = 0.002), and receiving multiple surgeries during the course of hospitalization (OR 32.25, 95%CI 18.21 to 57.11, P < 0.001).

## Conclusions

The perioperative incidence of VTE was substantially lower in this study than in previous reports for Western populations. Patients who were female, had ORIF and multiple surgeries were highly associated with the occurrence of VTE.
